# Supplementary material for: Are medications safely used by residents in elderly care homes? – A multi-centre observational study from Sri Lanka
Source: PLoS One. 2020 Jun 4;15(6):e0233486. doi: 10.1371/journal.pone.0233486 (PMC7272092; doi:10.1371/journal.pone.0233486)
Supplement: S1 Table — (DOCX) [file pone.0233486.s001.docx]

**S1 Table: Definitions and criteria used in the identification of medication errors**

| **Term** | **Definition** |
| --- | --- |
| **Medication error** | Any failure in the treatment process that may harm or has the potential to harm patients. |
| **In-house definitions of prescribing errors and subcategories of prescribing errors** | |
| **Prescribing errors** | Failures in the prescribing process that may harm or has the potential to harm patients |
| Wrong medicine | Includes prescribing duplications, unacceptable medicine combinations, clinically significant medicine interactions, potential adverse drug reactions and unnecessary medicine |
| Prescribing duplications | When two or more medicines of the same group being prescribed without a plausible explanation |
| Unacceptable medicine combinations | When two or more medicines which are not in the same group, but is unnecessary to be used concomitantly |
| Clinically significant medicine interactions | When two medicines in the same prescription had the potential to cause a known clinically significant interaction |
| Potential adverse drug reactions | Suspected adverse reactions  Avoid contraindications (Includes known allergies) |
| Unnecessary medicine | Though there is not a particular clinical condition a medicine has been prescribed |
| Medicine omissions | When a medicine which is needed (according to evidenced-based treatment guidelines) is omitted in the prescription or when a patient’s regular medicine has been omitted in the current prescription without a valid reason |
| Wrong dose | When the quantity of medicine recommended to be taken at a particular time is incorrect. |
| Wrong frequency | When the prescribed frequency of a medicine is different from current evidenced based treatment guidelines |
| Wrong duration | When the duration of a medication regimen is different from current evidenced-based treatment guidelines |
| Wrong dosage form | When the best available dosage form is not prescribed for a particular condition or when a dosage form not intended by the prescriber is written on the prescription E.g. Intravenous instead of intramuscular; when a dosage form that is not available for a medicine is prescribed E.g. oral insulin |
| **In-house definitions for medicine administration errors and subcategories of medicine administration errors** | |
| **Medicine administration error** | Failures in the medication administration process that may harm or has the potential to harm patients |
| Wrong dose | When a quantity of medicine different to the quantity recommended to be taken at a particular time in a prescription is administered |
| Wrong frequency | When a medicine frequency different to that of a prescription is administered |
| Wrong time | When a medicine is administered at a time different to that stated in the prescription |
| Medicine omission | When a prescribed medicine is not administered, intentionally skipped by patient/caregiver or slipped |
| **In-house definitions of dispensing errors and subcategories of dispensing errors** | |
| **Dispensing error** | Is a discrepancy between a prescription and the dispensing label dispensed with the relevant medicines by the pharmacy and dispensing of medicines with inferior pharmaceutical or informational quality |
| Wrong name | When a name different to what is prescribed in the prescription is written on the dispensing label of the relevant medicine |
| Wrong or missing dose | When a medicine dose different to what is prescribed in a prescription is written on the dispensing label of the relevant medicine or medicine/s prescribed are missing on the label |
| Wrong or missing frequency | When a medicine frequency different to that of a prescription is written on the dispensing label of the relevant medicine or no frequency has mentioned on the label |
| Wrong or missing duration | When a duration different to that of a prescription is written on the dispensing label of the relevant medicine or no duration has mentioned on the label |
| Wrong or missing dosage form | When a dosage form different to that of a prescription is written on the dispensing label of the relevant medicine or no dosage form has mentioned on the label |
| Wrong or missing the total number of units dispensed | When a total number of units different to that of a prescription is dispensed or a wrong number of units is written on the dispensing label of the relevant medicine |
| Wrong or missing essential directions to use medicines on a dispensing label | When the essential directions are written incorrectly or missing on the dispensing label  Ex: Writing as omeprazole once a day instead of writing 20 minutes before the breakfast |
| **In-house definitions of storage errors and subcategories of storage errors** | |
| **Storage errors** | Failures in the storage process (not maintaining the appropriate temperature, exposing to the sunlight, humidity or not in a ventilated area) that may harm or has the potential to harm patients |
| Suboptimal storage temperature or exposed to sunlight | When a medicine is not stored according to the temperature given in the label claim or exposed to direct sunlight |
| Use of inappropriate containers | When a medicine is not stored in an appropriate container/ stored in a container contrary to recommendations on the label claim |
| Inadequately separated from other medicines | When a medicine is not separated physically from other medicines |
