# Supplementary material for: Are medications safely used by residents in elderly care homes? – A multi-centre observational study from Sri Lanka
Source: PLoS One. 2020 Jun 4;15(6):e0233486. doi: 10.1371/journal.pone.0233486 (PMC7272092; doi:10.1371/journal.pone.0233486)
Supplement: S2 Table — (DOCX) [file pone.0233486.s002.docx]

**S2 Table:** **National Coordinating Council for Medication Error Reporting and Prevention (NCC MERP) Index for Categorizing Medication Errors used to categorize prescribing errors**

| **Severity of outcome** | **Description of outcome** | **Category** |
| --- | --- | --- |
| No Error | Circumstances or events that have the capacity to cause error | Category A |
| Error, No Harm | An error occurred but the error did not reach the patient (An "error of omission" does reach the patient) | Category B |
|  | An error occurred that reached the patient but did not cause patient harm | Category C |
|  | An error occurred that reached the patient and required monitoring to confirm that it resulted in no harm to the patient and/or required intervention to preclude harm | Category D |
| Error, Harm | An error occurred that may have contributed to or resulted in temporary harm to the patient and required intervention | Category E |
|  | An error occurred that may have contributed to or resulted in temporary harm to the patient and required initial or prolonged hospitalization | Category F |
|  | An error occurred that may have contributed to or resulted in permanent patient harm | Category G |
|  | An error occurred that required intervention necessary to sustain life | Category H |
| Error, Death | An error occurred that may have contributed to or resulted in the patient’s death | Category I |
