# Supplementary material for: Are medications safely used by residents in elderly care homes? – A multi-centre observational study from Sri Lanka
Source: PLoS One. 2020 Jun 4;15(6):e0233486. doi: 10.1371/journal.pone.0233486 (PMC7272092; doi:10.1371/journal.pone.0233486)
Supplement: S1 File — (DOCX) [file pone.0233486.s003.docx]

# S1 file Questionnaire on basic information and demographics of residents

QUESTIONNAIRE

1) Serial No: Date :

2) Name of the elders` home:

3) Inmate`s registration No:

4) Date of Admission:

5) Duration of patient stay in elders` home:

(From admission date to December 2016)

6) Medication administered by: Inmate Care giver

7) Age: years

8) Gender: Male Female

9) Education level: No schooling

Grade 1 to 5

Grade 6 to 11

Passed GCE O/L

Passed GCE A/L

Other

Specify: ……………………………..………………………………………………

10) Medical history (as medical records and verbal) :

|  | Current diseases |
| --- | --- |
| Verbal |  |
|  |  |
|  |  |
|  |  |
|  |  |
|  |  |
| Documented |  |
|  |  |
|  |  |
|  |  |
|  |  |
|  |  |

Table 5 -Medical history of inmate as per medical records and verbal (Non communicable chronic diseases)

11) Any known allergies to,

Medicines

Food

Plaster

None

If “yes” specify:………………………………………………………………………….
