# Supplementary material for: Are medications safely used by residents in elderly care homes? – A multi-centre observational study from Sri Lanka
Source: PLoS One. 2020 Jun 4;15(6):e0233486. doi: 10.1371/journal.pone.0233486 (PMC7272092; doi:10.1371/journal.pone.0233486)
Supplement: S2 File — (DOCX) [file pone.0233486.s004.docx]

# S2 file Checklist to assess the appropriateness of dispensing

# Appropriateness of label (Comparison of dispensed label with medical document)

| Name of medicine. | Right  Name | Right dosage form | Right  Strength | Right Number of units/  volume per dose | Right frequency | Right duration | Total No of units dispensed | Instructions | Remarks |
| --- | --- | --- | --- | --- | --- | --- | --- | --- | --- |
|  |  |  |  |  |  |  |  |  |  |
|  |  |  |  |  |  |  |  |  |  |
|  |  |  |  |  |  |  |  |  |  |
|  |  |  |  |  |  |  |  |  |  |
|  |  |  |  |  |  |  |  |  |  |
|  |  |  |  |  |  |  |  |  |  |
|  |  |  |  |  |  |  |  |  |  |
|  |  |  |  |  |  |  |  |  |  |
|  |  |  |  |  |  |  |  |  |  |
|  |  |  |  |  |  |  |  |  |  |
|  |  |  |  |  |  |  |  |  |  |
|  |  |  |  |  |  |  |  |  |  |

(✓ ,Yes ; 🗶 , No ; N/A , Not applicable)
