# Supplementary material for: Are medications safely used by residents in elderly care homes? – A multi-centre observational study from Sri Lanka
Source: PLoS One. 2020 Jun 4;15(6):e0233486. doi: 10.1371/journal.pone.0233486 (PMC7272092; doi:10.1371/journal.pone.0233486)
Supplement: S4 File — (DOCX) [file pone.0233486.s006.docx]

# S4 file Checklist to assess the appropriateness of medicine storage

**Appropriateness of storage (Direct observation)**

| Name of medicine. | Temperature  (optimum) | Not exposed to sunlight | Appropriate  container | Appropriate separation of each medicine type | Remarks |
| --- | --- | --- | --- | --- | --- |
|  |  |  |  |  |  |
|  |  |  |  |  |  |
|  |  |  |  |  |  |
|  |  |  |  |  |  |
|  |  |  |  |  |  |
|  |  |  |  |  |  |
|  |  |  |  |  |  |
|  |  |  |  |  |  |
|  |  |  |  |  |  |
|  |  |  |  |  |  |
|  |  |  |  |  |  |
|  |  |  |  |  |  |

b) Other inappropriate administration technique (Direct observation)

| Name of medicine. | Breaking of tablets  (Enteric coated, SR tablets) | Use of measuring devices for medicines | In accessibilities  Eg : Difficult to reach the bottle | Remarks |
| --- | --- | --- | --- | --- |
|  |  |  |  |  |
|  |  |  |  |  |
|  |  |  |  |  |
|  |  |  |  |  |
|  |  |  |  |  |
|  |  |  |  |  |
|  |  |  |  |  |
|  |  |  |  |  |
|  |  |  |  |  |
|  |  |  |  |  |
|  |  |  |  |  |
|  |  |  |  |  |
