# Supplementary material for: Are medications safely used by residents in elderly care homes? – A multi-centre observational study from Sri Lanka
Source: PLoS One. 2020 Jun 4;15(6):e0233486. doi: 10.1371/journal.pone.0233486 (PMC7272092; doi:10.1371/journal.pone.0233486)
Supplement: Attachment — Submitted filename: Reponse to Reviewers.docx [file pone.0233486.s009.docx]

**Response to reviewer comments**

***Reviewer 1****: The authors have addressed most of my concerns regarding the original version of the paper and made substantial improvements. However, there are still some minor revisions that I believe are necessary prior to publication. Specifically:*

***Comment 1***

***General***
*The entire manuscript should be reviewed in term of language punctuation*

**Response 1**

The manuscript was edited by a native English speaker previously and now being thoroughly reviewed by the authors.

***Comment 2
Introduction***
“Labour” change to ” labor”

Corrected (Please refer Line 62)

“Ageing” change to “aging”
Corrected (Please refer Line 55)

Line 57: are change to “is”

Corrected (Please refer Line 56)

***Comment 3***

*Line 69: ‘carers” should be replaced with a better word*

**Response 3**

Replaced with caregivers (Please refer Line 70)

***Comment 4***

*Line 71: United State (US)*

**Response 4**

Changed (Please refer Line 72)

***Comment 5***

*Line 90: “may be” change to “maybe”*

**Response 5**

Changed (Please refer Line 88)

***Comment 6***

*Line 90: “to” change to “from”*

**Response 6**

Changed (Please refer Line 89)

***Comment 7***

*Methods
Line 115: However,*

**Response 7**

Edited (Please refer Line 111)

***Comment 8***

*Result
Line 198:* *Eighty-five percent of residents*

**Response 8**

Edited (Please refer Line 191)

*Comment 9*

*1ine 203: most*

**Response 9**

Changed (Please refer Line 196)

***Comment 10***

*Discussion
Line 293: “carers” should be replaced with a better word*

**Response 10**

Replaced with the word “caregivers” (Please refer Line 288)

***Comment 11***

*Line 311: “household”*

**Response 11**

Edited (Please refer Line 305)

Reviewer 2:

Abstract
***Comment 1:*** *Line 43, administration error numbers were not normally distributed, so the median and 25th-75th centiles should be quoted. Please could the statistician also check that other parameters were normally distributed before quoting just the means and SDs.*

**Response 2**

Now we have shown both the mean (standard deviation) and median (25th-75th centiles) values in the abstract and the main text.

Introduction
***Comment 2:*** *Line 67. Please could you confirm that the social care workers were all unpaid? This would not be the case in the UK, so an explanation would be helpful.*

**Response 2**

Some of the social care workers at residential care homes work voluntarily and are not paid a formal salary while some are hired as paid staff. The sentence was re-worded as follows.

“Most of these facilities do not employ trained healthcare professionals but employ staff who have not received any formal training on safe use of medicines.”

***Comment 3:*** *Lines 80-83. It seems that there are conclusions in the introduction.*

**Response 3**

Thank you and we agree. Now we have removed this part from the introduction.

Sample size
***Comment 4:*** *This is a convenience sample. The calculation presented bears no relation to the outcomes investigated. I suggest remove the calculation and explain the factors limiting the study size.*

**Response 4**

Thank you and we agree. Now we have removed the description of the sample size calculation.

Sample selection
***Comment 5:*** *Line 14. Did the homes refuse access or consent?*

**Response 5**

The homes did not grant permission for researchers to approach the residents and thereby refused access. Now we have reworded it in the manuscript (Please refer Line 109).

***Comment 6:*** *How many residents were excluded because they were <60? How many actually refused?
Process*

**Response 6**

Unfortunately, we have not documented the demographics of those excluded nor the specific reason for exclusion. We only considered if participants matched the inclusion criteria. Hence, it is not possible to answer this query.

***Comment 7:*** *Line 137. Were these references the current editions at the time of the study? if so, need to state this.*

**Response 7**

Yes, the study was conducted in 2016 and the team used the current latest available editions at that time. The following was added,

“The current and latest available editions of British National Formulary (BNF) (Version 71) [26], the Australian Medicines Handbook (AMH) [27] and Medscape Pharmacists [28] were used as references for detecting prescribing errors” (Please refer Line 130).

.

Errors
***Comment 8:*** *Line 203. Need to say in what way the frequencies were incorrect.*

**Response 8**

Now we have discussed it (Please refer Line 196).

Discussion

***Comment 9:*** *Lines 343 et seq. to state that no harm occurred, the BPs and glucose concentrations need to be given. What checks were undertaken to ensure there were no signs and symptoms that might have attributable to the errors? If none, this might be a limitation of the study.*

**Response 9**

This observational study was carried out through reviewing documents and patient interview. As blood pressure values and glucose concentrations were not recorded on the patients’ medication documents, it was not possible to identify medication related patient harm. This has now been acknowledged under limitation (Please refer Line 334).

References
***Comment 10:*** *Some updating is needed e.g. annual health bulletin 2016 [2].*
Thank you however, in Sri Lanka, there is no health bulletin publication available after 2016.

Style
***Comment 11:*** *There are a few problems with English, which will need the attention of copy editors.*

**Response 11**

The manuscript was edited by a native English speaker previously and now being thoroughly reviewed by the authors.
