# Supplementary material for: Are medications safely used by residents in elderly care homes? – A multi-centre observational study from Sri Lanka
Source: PLoS One. 2020 Jun 4;15(6):e0233486. doi: 10.1371/journal.pone.0233486 (PMC7272092; doi:10.1371/journal.pone.0233486)
Supplement: Attachment — Submitted filename: Response to Reviewers.docx [file pone.0233486.s010.docx]

**Response to reviewer comments**

**Reviewer 1**: The quality of the paper considerably improved than the original version. I recommend this submission for acceptance with a minor revision: some lines and arrows are not straight in figure 1 that should be reformatted.

Thank you for the comment and now we have done the required changes in the Figure 1.

**Reviewer 2:**

Thank you for the opportunity to re-read this paper.

**Comment 1:** Copy editing is needed.

**Response 1:** Thank you and in addition to English editing done by Ms Cathy Lynch, a Clinical Pharmacist and a member of Collaborative of Australian Sri Lankan Pharmacy Practice Education Research (CASPPER) in the previous submission the entire manuscript has been revisited by Professor Lakshman Samaranayake, Professor Emeritus, University of Hong Kong , Hong Kong and copy edited the article thoroughly. We hope this effort is satisfactory.

**Comment 2:** I think it should be explained in the paper that some workers in care homes were unpaid, and worked voluntarily.

**Response 2:** Please refer line 68-70. This comment was already addressed in the previous submission but we have now further emphasized that most workers are unpaid and work on a voluntary basis.

**Comment 3:** The sample size should be based on the primary outcome. This does not appear to be the case. It could be removed, as explained previously.

**Response 3:** In your previous comments, you had advised us to remove the sample size calculation and discuss the limitations. Taking this comment into consideration, we had already removed the sample size calculation from the methodology and explained the factors limiting the study sample size. However, taking this comment into consideration again, we have further elaborated that the sample selected for study is a convenience sample. We have also re-worded the relevant paragraph for better clarity (Line 107 – 115).

**Comment 4:** The next text indicates that 5 of 34 homes refused access, and 9 were recruited. Is there a typographical error? Line 109

**Response 4:** It was not a typographical error. Of 34 elderly care homes registered, only 14 were functioning at the time of study. Out of the 14 functioning, 5 refused access and remaining 9 were approached. It is also mentioned in the manuscript text (Line 109 – 115).

**Comment** **5:** Failure to note reason for exclusion is a major limitation, and should be discussed.

**Response 5:** We have not recorded the individual reasons of the excluded sample, however, the overall elders who were excluded did not comply with at least one of our inclusion criteria. We have already acknowledged this in the limitation section (Line 342 -348). We have also acknowledged this limitation in the section dealing with sample size (Line 114-115) .

**Comment 6:** The case studies make this an interesting paper on an international topic that should be highlighted.

**Response 6:** Thank you for the comment and we believe no changes are required here.
